# Psychological Impact of Acne Vulgaris Among Young Females in the Eastern Province, Saudi Arabia

**DOI:** 10.7759/cureus.29378

**Published:** 2022-09-20

**Authors:** Abdul Sattar Khan, Abdullah F Almulhim, Maram H Alqattan, Noura F Almakhaitah, Fai I Alomair, Abdullah A Alkhateeb

**Affiliations:** 1 Department of Family and Community Medicine, King Faisal University, Riyadh, SAU; 2 Department of Family and Community Medicine, King Faisal University, Hufof, SAU

**Keywords:** kingdom of saudi arabia (ksa), quality of life, dlqi, psychological impact, female, acne vulgaris

## Abstract

Introduction

Acne vulgaris is considered one of the most common dermatological issues that affect approximately 9.4% of the world’s population, and the most affected group is adolescents. Acne can vary among adolescents and adults of different ethnicities and countries.

Aim

This study aimed to assess the psychological impact of acne vulgaris among female adults in the Eastern province of Saudi Arabia.

Subjects and methods

This is a cross-sectional study conducted among young females aged 15 to 25 years old. A self-administered validated questionnaire translated into Arabic was distributed among the female respondents with acne vulgaris using an online survey. The questionnaire includes basic demographic data and the Dermatology Life Quality Index (DLQI) questionnaire to assess the psychological impact of acne vulgaris.

Results

Four hundred seventy-six female participants aged 15-25 years took part. The majority were single (86.3%) and of Saudi nationality (92.4%). The prevalence of psychological impact affecting acne patients was 85.5% (ranging from low effect to severe effect). A significant relationship was observed between psychological impact according to marital status (p=0.034) and educational level (p=0.023). In a multivariate regression model, patients who had never been married and were more educated were the independent significant factors associated with increased rates of psychological impairment.

Conclusion

The quality of life of young females has been greatly affected by acne vulgaris. The severity of psychological impairment associated with acne vulgaris can be significantly predicted among educated and single females. Psychological counseling is necessary to decrease the burden caused by the dermatologic condition affecting young females in the region.

## Introduction

Acne is a long-term inflammatory condition of the pilosebaceous unit characterized by seborrhea, comedones, papules, pustules, nodules, cysts, and, in some cases, scars and keloids [1]. Acne on the face is common, exacerbating concerns about body image and sociability. As a result, it is not surprising that a person with visible acne may experience significant psychosocial difficulties [2]. Acne vulgaris is considered one of the most common dermatological issues, affecting approximately 9.4% of the world’s population; the most affected group is adolescents. Acne can vary among adolescents and adults of different ethnicities and countries [3,4]. Acne is reported to persist until the age of 20 years in about 64% of people and may even last for some individuals until they are 30 years old [3]. Acne has a significant impact on adults and older individuals of both genders. It can cause many problems, such as low self-esteem and low confidence [5]. Evaluation of acne based solely on clinical assessment does not consider the impact of the disease on individuals’ quality of life [6]. Several studies have been carried out worldwide to study the psychological impact of acne on patients. One study has reported anxiety, depression, and embarrassment among patients with acne [7]. Surprisingly, depression and anxiety are more prevalent among adults with acne than among adolescents. This is consistent with previous studies [8,9]. Adults with acne experience distress as a result of sociocultural assumptions that acne is uniquely a juvenile condition [8,9]. In Saudi Arabia, research into the psychological impact of acne vulgaris among female students found that nearly half of the participants had a mild impact [10]. Another study conducted in Tabuk City found that shyness and embarrassment were the most common impacts. There are not enough data available to estimate the psychological impact of acne among female adults, especially in this geographical area. This study aims to assess the psychological impact of acne vulgaris among female adults in the Eastern Province of Saudi Arabia.

## Materials and methods

In the Eastern Province, Saudi Arabia, a cross-sectional, Arabic-self-administered questionnaire-based study was conducted on young females selected by a simple random method. In prior investigations, the Dermatology Life Quality Index (DLQI) questionnaire has been validated [11]. The sample size was estimated at 500, using the recommended formula for the calculation of sample size for a cross-sectional study [12]. Only 15-25 years of age females were included who lived in the eastern region. The questionnaire was divided into two sections, the first of which contained biographical information about the patient, such as age, gender, nationality, and education. The second section of the DLQI questionnaire was used to measure the health-related quality of life of adult patients with skin problems. The ethical approval number (KFU-REC-2021-NOV-EA000226) has been taken from the ethics and research committee of the university.

Instrument and data collection

We distributed the questionnaire to female participants with acne and asked them to fill out the questionnaire of the Dermatology Life Quality Index (DLQI) to assess the impact of acne on quality of life (QoL). Using a validated DLQI Arabic version (Cronbach’s alpha coefficient was 0.8). The questionnaire consisted of 10 questions regarding disease symptoms, feelings, daily activities, type of clothing, social or physical activities, exercise, job or education, interpersonal relationships, marriage relationships, and treatment. The participants with acne were asked about problems faced during the previous week due to the acne, a duration considered to be easily and accurately recallable. Each question has a category of “Many times” (coded 3), “Often” (coded 2), “Sometimes” (coded 1), and “Never” (coded 0). The overall DLQI has a score ranging from 0 to 30. Higher scores indicate poor QoL. The effect of disease on QoL has been divided into five categories according to the severity: (0-1) without effect, (2-5) low effect, (6-10) moderate effect, (11-20) high effect, and (21-30) severe effect [13]. This has been reclassified into two categories: without effect and with effect (low, moderate, high, and severe effect levels).

Statistical analysis

Categorical variables were expressed as frequency and proportion (%), while continuous variables were expressed as mean and standard deviation. The relationship between the level of psychological impact according to the socio-demographic features of the female with acne vulgaris had been conducted using the Chi-square test. Then, significant results were tested in a multivariate regression analysis to determine the independent significant factor associated with the psychological impact of acne vulgaris where the odds ratio, as well as the 95% confidence interval, were also reported. A P-value below 0.05 was considered statistically significant, while a p-value of 0.01 was considered highly significant. All data analyses were performed using the Statistical Package for Social Sciences (SPSS) Version 26 (IBM Corporation, Armonk, New York, USA).

## Results

In total, 476 female participants responded and fulfilled the inclusion criteria, which makes the response rate 95%. Table 1 presents the socio-demographic characteristics of female respondents. Nearly all respondents were females (86.3%), with nearly one-third (32.8%) living in Dammam, mostly being Saudi (92.4%). Concerning education, 60.1% had a diploma or below degree.

**Table 1:** Socio-demographic characteristics of female participants (n=476).

Study variables	No	%
Marital status		
Single	411	86.3%
Married	59	12.4%
Divorced	6	1.3%
Residential location		
Al Ahsa	134	28.15%
Dammam	156	32.77%
Al Khobar	75	15.75%
Jubail	37	7.75%
Qatif	68	14.%
Hafr Al Batin	6	1.3%
Educational level		
Diploma or below	286	60.1%
Bachelor or above	190	39.9%
Nationality		
Saudi	440	92.4%
Non-Saudi	36	7.6%

**Figure 1:**
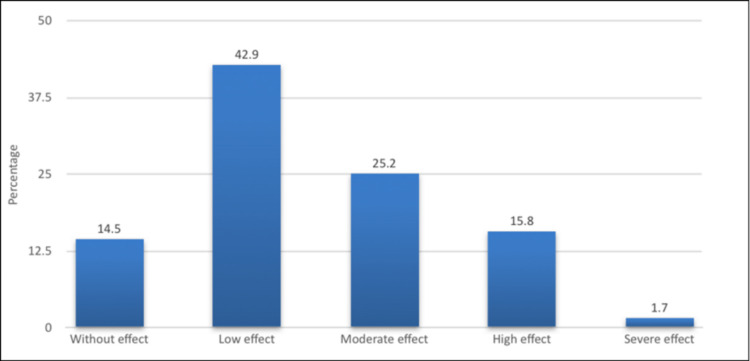
Severity of psychological impact due to acne vulgaris.

The assessment of the psychological impact due to acne vulgaris is given in Table 2. According to the results, it was observed that the mean score of DLQI was 6.00 (SD 4.9). Approximately 85.5% of the respondents experienced psychological impairment due to acne vulgaris and only 14.5% were normal. Regarding the severity of quality of life, low, moderate, high, and severe compromise 42.9%, 25.2%, 15.8%, and 1.7%, respectively (see Figure 1).

**Table 2:** Descriptive statistics of the Dermatology Quality of Life Index (DLQI) questionnaire (n=476).

DLQI criteria	No	%
DLQI total score (mean ± SD)		
4.89 ± 6.00		
Level of psychological impact		
With effect	407	85.5%
Without effect	69	14.5%
Severity of psychological impact		
Without effect	69	14.5%
Low effect	204	42.9%
Moderate effect	120	25.2%
High effect	75	15.8%
Severe effect	8	1.7%

When measuring the rate of psychological impact according to the basic demographic characteristics of the female with acne vulgaris, it was found that there was a significant relationship between psychological impact among marital status (p=0.034) and educational level (p=0.023), while the differences between residence location (p=0.662) and nationality (p=0.700) did not reach statistical significance (see Table 3).

**Table 3:** Level of psychological impact based on the socio-demographic characteristics of the female with acne vulgaris (n=476). P-value has been calculated using the Chi-square test. **Significant at p<0.05 level.

Factor	Psychological impact				p-value
	With effect		Without effect		
	No (n=407)	%	No (n=69)	%	
Marital status					0.034**
Never been married	357	87.7%	54	78.3%	
Been married	50	12.3%	15	21.7%	
Residence location					0.662*
Al Ahsa	119	29.2%	15	21.7%	
Dammam	133	32.7%	23	33.3%	
Al Khobar	61	15%	14	20.3%	
Qatif	57	14%	11	15.9%	
Jubail or Hafr Al Batin	37	9.7%	6	8.7%	
Educational level					0.023**
Diploma or below	236	58%	50	72.5%	
Bachelor or above	171	42%	19	27.5%	
Nationality					0.700
Saudi	377	92.6%	63	91.3%	
Non-Saudi	30	7.4%	6	8.7%	

A multivariate regression model was applied to determine which factors were independently associated with psychological impairment. Based on the results, it was revealed that marital status and educational level were the factors independently associated with increased rates of psychological impairment. This further suggests that the risk of the females who have never been married is predicted to increase the risk of psychological impairment by at least twofold than those who had been married (AOR=2.075; 95% CI=1.082-3.980; p=0.028). Females who were more educated were more likely to increase the risk of psychological impairment by at least 1.96 times higher compared to those who were less educated (AOR=1.965; 95% CI=1.114-3.467; p=0.020) (see Table 4).

**Table 4:** Multivariate regression analysis to ascertain the effect of acne vulgaris psychologically among female respondents (n=476). AOR: adjusted odd ratio; CI: confidence interval.

Factor	AOR	95% CI	P-value
Marital status			
Never been married	2.075	1.082–3.980	0.028
Been married	Reference value		
Educational level			
Diploma or below	Reference value		
Bachelor or above	1.965	1.114–3.467	0.020

## Discussion

This study investigated the impact of acne vulgaris on the quality of life among young females in the Eastern Province of Saudi Arabia. The findings of this study revealed that the quality of life of our young females was largely affected by acne vulgaris. Approximately 85.5% of the respondents reported psychological impairment; among them, 42.9% had a low effect, 25.2% had a moderate effect, and 15.8% had a high effect. Only 1.7% were classified as severe. This is almost consistent with the paper of Alanazi et al. [10], in which 29% of female secondary school students indicated that acne vulgaris does not affect them psychologically. However, 56.3% reported a small to moderate impact (56.3%), and 14.5% had a large impact. In Tabuk City, Saudi Arabia [11], 33.5% of the young female students showed a low psychological impact. However, 20.4% of them claimed that shyness and embarrassment due to acne were the major psychological issues affecting their self-esteem and self-reliance. In Riyadh [14], a considerable proportion (40%) of the young population reported that acne vulgaris had no psychological impact on them, which was higher than in our research. Only 19% claimed to have a moderate effect, 9% had a large effect, and fewer than 1% had an extremely large effect. However, in Oman [15], more than half of the university students (52%) were shown to have impaired QoL due to this type of skin condition. Also, the majority of students (73%) complained of stress associated with acne, and 32% resorted to counseling to address acne-related stress. A good understanding of how psychological disorders play an important role in the exacerbation of skin conditions is important to providing early intervention to improve a patient’s quality of life: “Understanding the codes of adolescents today allows us to optimize the medical approach to acne associated with psychological conditions and facilitates treatment compliance and adherence” [16].

The results of our study further suggest that marital status showed a significant association with psychological impact, indicating that young females who had never been married tended to exhibit severe psychological impairment compared to females who had been married. Similarly, we noted that educational level had directly influenced the impairment of the Dermatology Life Quality Index: young females who were more educated showed poorer QoL than females who were less educated. This is contradicted by Alqahtani et al. [14], who found that the severity of psychological impact was more prevalent among less-educated participants and those with a longer duration of skin disease. However, our results showed that neither residence, location, nor nationality were relevant factors of psychological impact. On the other hand, several studies relate the severity of acne to psychological impairment, suggesting that the impairment of QoL increases with the increased severity of acne [10,17-19]. Contrary to these reports, Cherukuri et al. [20] found no correlation between impaired QoL and the severity of acne, implying that impairment of QoL was not necessarily related to the severity of acne, but it is worth considering the disability it causes when personalizing therapeutic management. The psychological state associated with acne has been well discussed in many studies. In particular, adolescents who complained of facial acne had a burden of negativity that complicated their daily lives physically, emotionally, and socially. Although the literature recognizes acne as an aggravating factor in young people’s psychological issues, it is assumed that this effect is magnified by the psychosocial evolution of adolescents in the current century [16].

Wherein embarrassment has been the major issue being faced by the young, specifically when attending social activities that greatly affect their self-esteem and self-confidence, leading to a greater impact on their quality of life [11].

## Conclusions

The quality of life of young females has been greatly affected by acne vulgaris. The severity of psychological impairment associated with acne vulgaris can be significantly predicted among educated and single females. Psychological counseling is necessary to decrease the burden caused by this dermatologic condition affecting young females in our region. Timely treatment of acne could result in instantaneous relief of the skin condition leading to a better quality of life. Further research is needed to get more insights into the quality of life of young females with underlying skin diseases. Though we have tried to reduce bias as much as possible, there are a few limitations to study. We can include more data relevant to family support, peer reaction, and duration of the condition.
